# Engineered PS and PAN Microplastic Fibers Elicit Stronger A549 Epithelial Responses than Spherical Polystyrene Particles

**DOI:** 10.1021/acs.chemrestox.6c00243

**Published:** 2026-08-05

**Authors:** Sewoon Kim, David M. Cwiertny, Dongkeun Lee, Jong Sung Kim, Jon A. Doering, Qinglin Wu

**Affiliations:** † Department of Civil and Environmental Engineering, 5779 Louisiana State University, Baton Rouge, Louisiana 70803, United States; ‡ Department of Civil and Environmental Engineering, 4083 University of Iowa, Iowa City, Iowa 52242, United States; § Department of Chemistry, University of Iowa, Iowa City, Iowa 52242, United States; ∥ Department of Occupational and Environmental Health, College of Public Health, University of Iowa, Iowa City, Iowa 52242, United States; ⊥ Department of Environmental Sciences, College of the Coast & Environment, Louisiana State University, Baton Rouge, Louisiana 70803, United States; # School of Renewable Natural Resources, LSU AgCenter, Baton Rouge, Louisiana 70803, United States

## Abstract

Polymeric fibers are a prevalent form of airborne micro- and nanoplastics (MNPs), yet their inhalation hazards remain poorly constrained because toxicological studies have largely relied on spherical particles or heterogeneous fiber materials. Here, polystyrene (PS) and polyacrylonitrile (PAN) fiber MNPs were fabricated by electrospinning followed by cryomilling, probe sonication, and filtration, yielding discrete fibers with comparable diameters and lengths. FTIR confirmed preservation of polymer identity and showed no detectable residual solvent signal in the isolated fibers. Using A549 lung epithelial cells as an inhalation-relevant in vitro model, we found that PS and PAN fiber MNPs induced dose-dependent reductions in viability together with increased membrane damage, oxidative stress, and IL-8 release, whereas spherical PS particles elicited comparatively limited responses at the tested mass concentrations. Scanning electron microscopy further showed abnormal cell–fiber interactions, including membrane deformation and apparent partial membrane wrapping around fibers, consistent with morphology-dependent cellular injury. Together, these results show that engineered polymeric fiber MNPs elicit stronger epithelial stress and inflammatory responses than spherical particle controls under the tested conditions, supporting morphology-aware evaluation of airborne MNP hazards.

## Graphical abstract

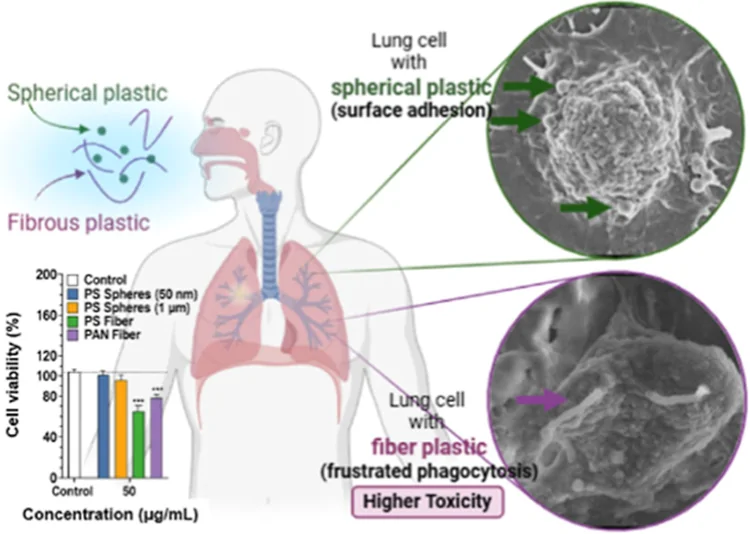

## Introduction

Polymeric fibers are extensively used in textiles, filtration media, and a wide range of industrial applications because of their favorable mechanical properties, durability, and low production cost.^1–3^ As a result of their widespread use, increasing evidence indicates that polymeric fibers are continuously emitted into the environment through atmospheric release and deposition,^4,5^ wastewater effluent,^6,7^ and the degradation of consumer products.^8,9^ Recent studies further demonstrate that fiber-shaped micro- and nanoplastics (MNPs) are the dominant form of airborne plastics in both outdoor and indoor environments, highlighting inhalation as a potentially important exposure pathway for humans.^10–13^ Particularly, Dris et al., reported substantial deposition of fiber-type of MNPs in indoor air sampled from private apartments and office environments, with deposition rate ranging from approximately 1586 to 11,130 fibers m^–2^ day^–1^.^11^ Because individuals spend approximately 90% of their time indoors, according to the U.S. Environmental Protection Agency,^14^ such measurements highlight the relevance of indoor airborne fibers as a potential and sustained pathway for human exposure.

These fibers differ fundamentally from spherical MNPs in their geometry, rigidity, and persistence. Their high aspect ratios and limited flexibility resemble characteristics of other pathogenic fibers, suggesting that polymeric fibers may trigger fiber-specific toxicological responses associated with physical cell–fiber interactions, oxidative stress, and inflammation.^15–17^ Despite these concerns, the majority of toxicology studies on MNPs to date have focused on spherical particles, which often exhibit limited toxicity,^18^ except when particles carry sorbed contaminants or additive chemicals.^19,20^ Consequently, the potential hazards associated with fiber-shaped MNPs remain insufficiently characterized, particularly with respect to how fiber physicochemical properties influence biological responses and potential toxicities.

Critically, while the toxicology of fiber-shaped materials such as asbestos and other mineral fibers has been extensively studied,^21,22^ comparatively few studies have systematically investigated polymeric fibers relevant to environmental exposure using controlled structural properties. Given the dominance of polymeric fibers in airborne MNPs, the lack of toxicological data derived from polymeric fibers with well-defined diameter, length, and rigidity represents a major barrier to accurate risk assessment. This gap is further exacerbated by the fact that many existing polymeric fiber studies rely on heterogeneous, commercially sourced, or mechanically fragmented materials with poorly controlled or uncharacterized geometries, limiting the ability to isolate the specific role of fiber geometry in driving biological responses. Consequently, the development of fiber-conscious material design and regulatory frameworks remains constrained.

Here, we present a fabrication-enabled toxicological framework for polymeric fiber MNPs, in which fibers were systematically engineered and evaluated for their biological responses, enabling assessment of geometry-dependent effects across different polymers. Polystyrene (PS) and polyacrylonitrile (PAN) were selected to balance environmental relevance with experimental control over fiber fabrication. PAN was chosen because it is a representative acrylic-fiber polymer and a well-established electrospinning material that enables reproducible preparation of fibers with tunable morphology.^23^ PS was selected because it is a common plastic polymer and a widely used reference material in microplastic toxicology.^24^ Importantly, including both PS fibers and commercially available PS spheres allowed direct comparison of morphology-dependent responses under the same polymer chemistry, helping distinguish shape effects from polymer-specific effects. We hypothesize that, at comparable mass and chemistry, fiber-shaped MNPs induce stronger cytotoxic, oxidative, and pro-inflammatory responses than spherical MNPs. Using human lung epithelial cells as an inhalation-relevant model, we compare engineered polymeric fibers with defined dimensions to commercially available spherical MNPs. This integrated fabrication–toxicity approach supports a morphology-aware framework for evaluating airborne polymeric fiber MNPs.

## Materials and Methods

### Fabrication of Fiber MNPs with Controlled Physicochemical Properties

All chemical compositions and electrospinning procedures are described in detail in the Supporting Information (SI). Briefly, PS and PAN nanofiber sheets were fabricated by electrospinning polymer solutions under controlled environmental conditions to produce sheets of continuous fibers with well-defined diameters and uniform morphology. The fabricated nanofiber sheets were then mechanically processed to obtain discrete fibers via cryomilling (Spex 6875, Cole-Parmer) followed by probe sonication (Digital Sonifier 450, Branson). Cryomilling was conducted with a precooling time of 2 min, run time of 2 min, and cool time of 2 min for four cycles at 15 counts s^–1^. The resulting fibers were dispersed in deionized (DI) water and subjected to probe sonication using 1 min on–1 min off cycles for a total of 30 min at 50% amplitude to achieve a homogeneous suspension. The suspension was subsequently passed through a 30 μm woven nylon filter (Spectra/Mesh Woven Filters) to remove incompletely milled or excessively long fibers. Prior to use, the filter was rinsed with 1 L of deionized (DI) water to remove any potential contaminants. Lastly, to determine the fiber weight in the prepared suspensions, the suspensions were ultracentrifuged at 150,000 *g* for 90 min. After removal of the supernatants, the recovered fibers were dried at 60 °C for 24 h. The dried fiber mass was then measured and used to prepare stock suspensions for toxicity evaluations at the desired concentrations.

### Characterization of Fabricated Polymeric Fiber MNPs

Fiber morphology was examined using scanning electron microscopy (SEM; S-4800, Hitachi). A drop of the fiber suspension was deposited onto a polycarbonate membrane (0.22 μm pore size, GTTP, Millipore) and dried prior to SEM imaging. Fiber diameter and length distributions were quantified from SEM micrographs using ImageJ software. Fiber chemical characteristics were analyzed by Fourier-transform infrared spectroscopy (FTIR; VERTEX 70 V, Bruker) to verify polymer composition and assess the presence of residual solvent species.

### Preparation of Polymeric Fiber MNP Suspensions and *In Vitro* Toxicity Assessment

Polymeric fiber MNPs were dispersed using serum proteins as dispersants following our previous protocol.^22^ Briefly, fibers were suspended in DI water containing fetal bovine serum (FBS), sonicated to reduce agglomeration, and diluted in complete RPMI 1640 medium prior to exposure. Human alveolar epithelial A549 cells were used as an *in vitro* pulmonary model to assess cytotoxicity of airborne polymeric fiber MNPs.^25,26^ To evaluate shape-dependent responses, commercial PS spheres (diameter size of 50 nm and 1 μm) were included as reference particles. Cells were exposed to polymeric fibers or PS spheres at concentrations of 12.5–50 μg/mL for 24 h. Following exposure, cell viability, oxidative stress, membrane integrity, and pro-inflammatory responses were assessed using standard fluorescence- and absorbance-based assays. All experiments were conducted in biological triplicates (*n* = 3), and statistical significance was evaluated using standard parametric tests (*p* < 0.05). Detailed experimental and statistical procedures are provided in the Supporting Information.

## Results and Discussion

### Characteristics of Fabricated Polymeric Fiber MNPs

Cryomilling followed by probe sonication effectively fragmented electrospun PS and PAN nanofiber sheets into discrete fiber MNPs. As shown in Figure 1, the resulting PS and PAN fibers exhibited well-defined length and diameter distributions while retaining cylindrical geometry and smooth surfaces. The fragmentation process primarily shortened fibers without inducing melting, fusion, or morphological deformation, demonstrating successful generation of discrete polymeric fiber MNPs suitable for subsequent toxicological evaluation.

**Figure 1:**
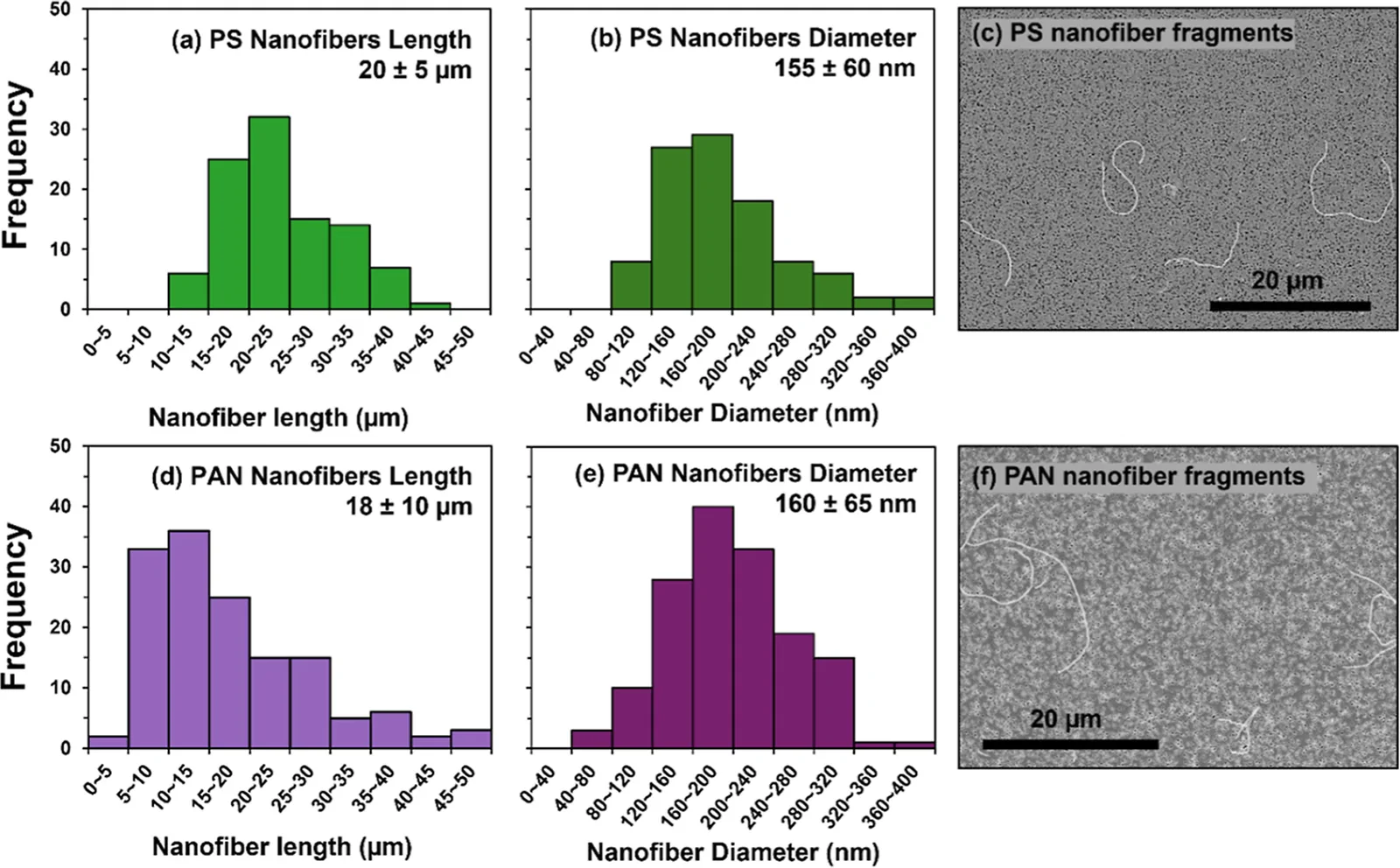
Size distributions and morphology of fabricated polymeric fiber MNPs. (a,d) Length and (b,e) diameter distributions of fragmented polystyrene (PS, green) and polyacrylonitrile (PAN, purple) fibers obtained after cryomilling, probe sonication, and filtration. Measurements were conducted on >100 individual fibers per polymer using ImageJ software. (c,f) Representative SEM images show discrete, well-separated fibers with smooth surfaces and preserved cylindrical morphology. PS and PAN fibers exhibit comparable length and diameter distributions following mechanical fragmentation.

Quantitative image analysis of more than 100 fibers per polymer (Figure 1) revealed that fabricated PS fibers exhibited mean lengths of 20 ± 5 μm and diameters of 155 ± 60 nm, while PAN fibers showed comparable lengths (18 ± 10 μm) and diameters (160 ± 65 nm). The narrow size distributions obtained after 30 μm filtration indicate effective removal of excessively long fragments and the generation of homogeneous fiber populations. The similarity in geometric characteristics between PS and PAN fibers further suggests that polymer chemistry did not substantially influence fragmentation behavior under the applied cryomilling and sonication conditions. The fabricated fibers are shorter than many textile-derived airborne fibers reported in environmental monitoring studies, which often detect fibers from tens to hundreds of micrometers or larger.^11,27^ However, reported airborne MNP size distributions are strongly influenced by sampling and analytical detection limits, and smaller fibrous particles are likely underrepresented in current data sets.^28^ Therefore, the PS and PAN fibers used here should be interpreted as controlled, respirable model fibers for testing morphology-dependent toxicity, rather than as materials intended to capture the full environmental size spectrum. Although a diameter-matched PS sphere would provide an additional stringent shape control, the 50 nm and 1 μm PS spheres were selected as commonly used nano- and microscale reference particles that bracketed the fiber diameter and allowed comparison across spherical particle sizes.^24^

The efficiency of the mechanical breakdown process is further illustrated in Figure S1. The original electrospun nanofiber sheets and their SEM images (Figure S1a,b) show continuous, randomly entangled morphologies, whereas cryomilling produced loose powder-like aggregates with no residual sheet structure (Figure S1c,d). Subsequent dispersion of the cryomilled powder in DI water followed by sonication resulted in a visually homogeneous fiber suspension and complete elimination of residual sheet morphology (Figure S1e). Representative SEM images of the fragmented materials collected on membrane filters (Figure S2) confirm the presence of individual, well-dispersed PS and PAN fibers suitable for in vitro exposure studies.

Length–diameter mapping (Figure S3) showed overlapping distributions for PS and PAN fibers without distinct clusters or long tails, indicating polymer-independent fragmentation and minimal aggregation during processing. Importantly, no correlation between fiber length and diameter was observed which further supports the absence of aggregation and the maintenance of discrete fibers in suspension. Preserving individual fibers is critical for evaluating aspect-ratio-dependent cellular responses, as aggregation can alter effective exposure dose and obscure intrinsic toxicity mechanisms.^29,30^

Chemical integrity of the fabricated fiber MNPs was confirmed by FTIR spectroscopy (Figure S4). The spectra of PS and PAN fibers closely matched those of their corresponding pristine polymers, displaying characteristic aromatic C–H stretching at 3060 cm^–1^ and C=C stretching bands at 1600 cm^–1^ for PS^31,32^ and the nitrile (C≡N) stretch at 2240 cm^–1^ for PAN.^33–35^ No new absorption features indicative of oxidation or polymer backbone modification were observed, and solvent-related peaks associated with DMF were absent, confirming complete solvent removal during electrospinning and postfabrication drying. Because FTIR spectra of the isolated fibers closely matched the corresponding pristine polymers and did not show detectable residual DMF features, the observed cellular responses are most reasonably interpreted in relation to the fabricated fiber materials rather than obvious residual solvent contamination.

Collectively, these morphological and spectroscopic analyses demonstrate that the fabrication and mechanical fragmentation strategy reproducibly yields chemically unmodified polymeric fiber MNPs with consistent geometries. SEM observations of the dispersed fiber suspensions showed predominantly discrete, well-separated fibers with no obvious large agglomerates, suggesting that the FBS-assisted sonication protocol limited aggregation prior to exposure. These materials therefore provide a suitable platform for comparative toxicological evaluation of PS and PAN fiber MNPs while minimizing variability associated with fiber morphology or processing-induced artifacts. However, quantitative time-resolved dispersion stability in complete exposure medium was not assessed, and dynamic aggregation or sedimentation during the exposure period cannot be excluded.

### Morphology-Dependent Cytotoxic, Oxidative, and Inflammatory Responses to Polymeric Fiber MNPs

Potential assay interference was evaluated using material-only controls for the Alamar Blue and LDH assays and IL-8 adsorption experiments. The tested materials showed minimal interference with the Alamar Blue and LDH assays, with signal recovery ranging from approximately 95–102% of reagent-only controls. IL-8 recovery remained above 89% for all materials, indicating limited cytokine adsorption under the experimental conditions. These results suggest that assay interference and cytokine adsorption were unlikely to materially influence the observed biological responses (Table S1). Across all tested concentrations, PS sphere MNPs elicited minimal biological responses. Cell viability remained above 90% even at 50 μg/mL, indicating limited acute toxicity from spherical PS particles. In contrast, both PS and PAN fiber MNPs induced a pronounced, dose-dependent reduction in viability, reaching approximately 60–70% of control levels at the highest concentration. Notably, this divergence occurred despite identical polymer chemistry in the case of PS spheres versus PS fibers, supporting an important role for particle morphology under the tested mass-normalized exposure conditions (Figure 2a).

**Figure 2:**
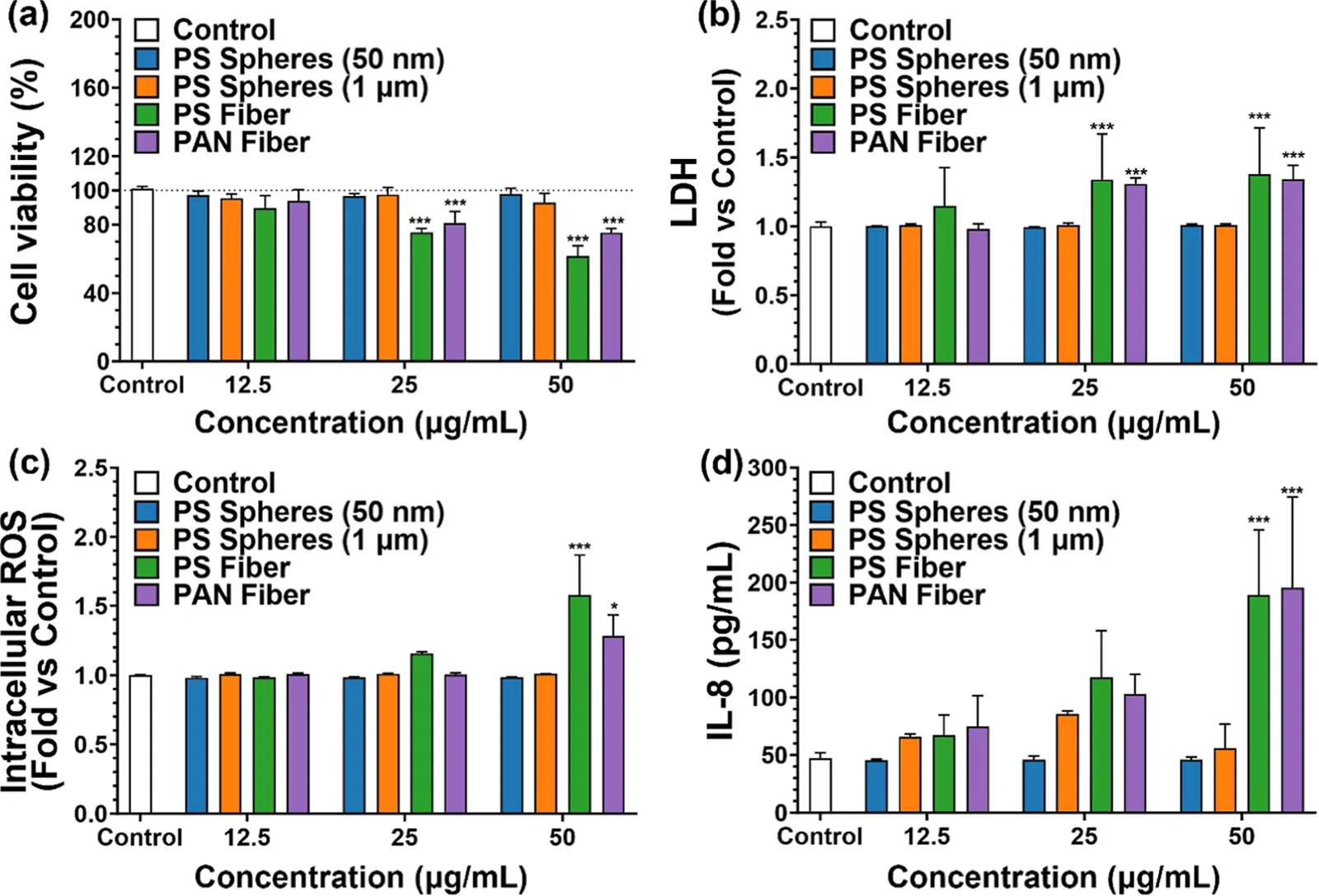
Shape-dependent cytotoxic, oxidative, and inflammatory responses in A549 cells exposed to polymeric fiber MNPs. A549 cells were exposed for 24 h to PS spheres (50 nm and 1 μm), PS fibers, or PAN fibers at the indicated concentrations (12.5–50 μg/mL). (a) Cell viability assessed by the Alamar Blue assay and expressed as percentage of the control. (b) Lactate dehydrogenase (LDH) release as an indicator of membrane damage, expressed as fold change relative to the control. (c) Intracellular reactive oxygen species (ROS) generation quantified using the DCFH-DA assay and expressed as fold change relative to the control. (d) IL-8 secretion in cell culture supernatants quantified by ELISA and expressed as unnormalized supernatant concentration in pg/mL. Data are presented as mean ± SD (*n* = 3). Statistical significance relative to the control was determined by one-way ANOVA followed by Tukey’s post hoc test (*p < 0.05, **p < 0.01, ***p < 0.001).

Consistent with reduced cell viability, exposure to fiber MNPs significantly increased lactate dehydrogenase (LDH) release, reflecting compromised membrane integrity. PS fiber MNPs induced an approximately 1.7-fold increase in LDH release, while PAN fiber MNPs produced an approximately 1.6-fold increase relative to untreated controls. Both PS sphere sizes did not significantly alter LDH levels. These findings suggest that elongated fibers impose greater mechanical stress on the plasma membrane, leading to localized damage and leakage. Similar shape-driven membrane disruption has been reported for other high-aspect-ratio materials, including carbon nanotubes and inorganic fibers, where elongated geometry increases the likelihood of physical membrane perturbation (Figure 2b).^36^

Oxidative stress emerged as a key mechanistic feature of fiber-induced toxicity.^37^ ROS levels increased substantially following exposure to PS fiber MNPs, reaching approximately 1.5–2-fold above control levels, while PAN fiber MNPs elicited a comparable but slightly attenuated response (Figure 2c). In contrast, neither 50 nm nor 1 μm PS spheres significantly affected ROS generation. Elevated ROS can disrupt redox homeostasis, impair mitochondrial function, and reduce cellular metabolic activity, which may partly explain the lower Alamar Blue signal observed in fiber-exposed cells.^38^ Thus, the ROS and viability results should be interpreted as related end points rather than independent responses. In the present study, increased ROS likely reflects a combination of membrane damage and prolonged cell–fiber interactions, rather than chemical reactivity intrinsic to the polymer. However, because ROS inhibition experiments were not performed, these data support a correlation between oxidative stress and reduced metabolic activity, but do not establish ROS as the sole causal driver of reduced viability.

The pro-inflammatory response closely paralleled oxidative stress responses. IL-8 secretion increased dramatically following fiber exposure, reaching levels approximately 4-fold above control levels at 50 μg/mL for both PS and PAN fibers, whereas PS spheres elicited minimal IL-8 induction (Figure 2d). IL-8 values are reported as unnormalized supernatant concentrations because all treatments were conducted using the same initial cell seeding density, culture volume, and exposure duration. Cell number and total protein were not measured after exposure; therefore, per-cell or per-protein IL-8 normalization was not performed. The IL-8 results should therefore be interpreted as total extracellular cytokine burden under each exposure condition and considered together with the viability, LDH, and ROS end points. IL-8 plays a central role in neutrophil recruitment and amplification of inflammatory responses in the lung, suggesting that polymeric fibers may promote inflammatory responses following inhalation exposure. Comparable IL-8 elevations have been observed in epithelial and macrophage models exposed to pathogenic fiber-shaped materials, supporting the relevance of fiber geometry in pulmonary inflammation. However, although the present study demonstrates coordinated changes in epithelial viability, membrane integrity, oxidative stress, and IL-8 secretion following exposure to polymeric fiber MNPs, the molecular signaling pathways underlying these responses were not directly investigated. Therefore, these findings should be interpreted as evidence of morphology-associated epithelial injury and inflammatory response rather than as evidence of a specific molecular mechanism. Future studies incorporating pathway-level end points, including NF-κB activation, Nrf2/HO-1 oxidative stress signaling, mitochondrial dysfunction, and lysosomal damage, will be important to determine the mechanisms underlying polymeric fiber-induced toxicity.^39^

Although concentration-dependent trends were observed across several end points, the present exposure design included a limited number of concentration points and was not intended for formal nonlinear dose–response modeling or EC50 estimation. Additional exposure concentrations spanning a broader response range would be required to derive robust potency parameters. Furthermore, while viability, LDH release, ROS production, and IL-8 secretion identify cytotoxic, membrane-damage, oxidative-stress, and inflammatory responses, additional pathway-level assays, such as NF-κB activation, Nrf2/HO-1 signaling, mitochondrial dysfunction, or lysosomal damage, would be needed to define the downstream mechanism more precisely.

### SEM Evidence of Cell–Fiber Interactions and Membrane Deformation

SEM imaging provides direct insight into the biochemical responses observed in Figure 2 by revealing distinct cell–material interaction modes. The 1 μm PS spheres were predominantly associated with the cell surface without overt membrane deformation or penetration (Figure 3b), consistent with the minimal cytotoxicity and inflammatory signaling induced by spherical particles. PS fibers, however, exhibited markedly different interactions with cells. Fibers frequently appeared to penetrate or span the plasma membrane, with partial internalization and fiber ends protruding from the cell surface (Figure 3c). The SEM images revealed membrane deformation and close fiber association with the epithelial cell surface, suggesting prolonged physical interactions between elongated fibers and epithelial cells. Because A549 cells are epithelial cells rather than professional phagocytes, these observations should not be interpreted as direct evidence of frustrated phagocytosis. Instead, they indicate that elongated fibers can produce abnormal physical cell–material contact, including membrane deformation and incomplete engulfment-like interactions. By comparison, spherical particles fail to elicit comparable responses because their geometry allows surface association or accommodation without substantial membrane deformation, limiting sustained stress signaling and downstream inflammatory activation. Together, these results support an important role for particle morphology in shaping cell–material interaction outcomes in this epithelial model.

**Figure 3:**
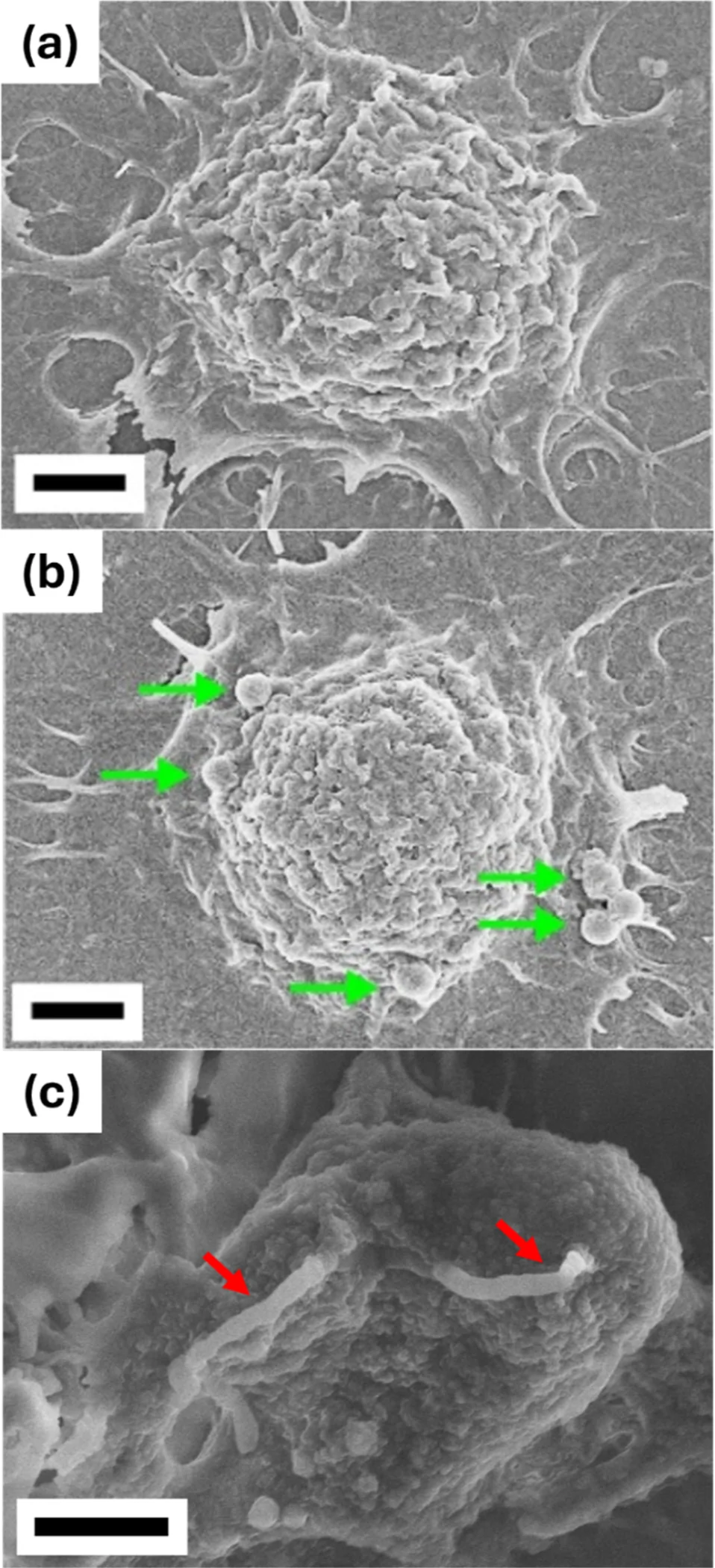
Shape-dependent interactions between polymeric materials and A549 cells observed by SEM. SEM images of A549 cells following 24 h exposure to polymeric materials. (a) Untreated control cells exhibiting intact surface morphology. (b) Cells exposed to 1 μm PS spheres, showing particle attachment on the cell surface (green arrows) without apparent membrane penetration or deformation. (c) Cells exposed to PS fibers show abnormal cell–fiber interactions, including membrane deformation, close fiber association, and apparent partial membrane wrapping around fibers (red arrows). These SEM observations indicate close physical contact between elongated fibers and A549 epithelial cells but should not be interpreted as direct evidence of macrophage-type frustrated phagocytosis. Scale bars represent 2 μm in all panels.

The observed cell–fiber interactions may contribute to the elevated LDH release, ROS generation, and IL-8 secretion observed following fiber exposure (Figure 2). SEM images demonstrated membrane deformation and close association between elongated fibers and the epithelial cell surface, suggesting that fiber geometry promotes prolonged physical interactions with epithelial cells. However, the present study did not directly investigate cellular uptake pathways or the mechanisms underlying these interactions, and SEM alone cannot distinguish among surface attachment, incomplete membrane accommodation, and cellular internalization. Therefore, the observed associations should be interpreted as descriptive evidence of morphology-dependent cell–fiber interactions rather than direct evidence of a specific uptake or phagocytic mechanism. This mechanism parallels established paradigms in asbestos and carbon nanotube toxicology, where high-aspect-ratio, rigid fibers resist complete engulfment, leading to persistent oxidative stress and inflammation.^40^ In this context, future studies could include well-characterized high-aspect-ratio materials, such as carbon nanotubes or asbestos fibers, as positive benchmark controls to calibrate the magnitude of polymeric fiber-induced responses. The present findings are consistent with the view that fiber-induced epithelial injury in this system is strongly influenced by physical cell–fiber interactions under the tested conditions.

Fiber rigidity may also contribute to the observed cell–fiber interactions. The elastic moduli of the fabricated PS and PAN fibers were not directly measured in this study, so the role of stiffness cannot be resolved quantitatively. However, reported literature values indicate that PS is a glassy polymer with Young’s modulus values typically in the low-GPa range, whereas electrospun PAN nanofibers can be stiffer depending on fiber diameter, molecular orientation, and processing history.^41–43^ A more rigid fiber would be expected to bend less during cell contact and may therefore promote membrane deformation or incomplete internalization. Because PS and PAN fibers had similar dimensions and produced broadly similar biological response patterns, the present results point to elongated geometry as a major shared driver of toxicity, while polymer-specific stiffness may still contribute to differences in response magnitude.

### Environmental Implications and Future Research Needs

This study provides controlled evidence that polymeric fiber morphology is associated with enhanced cytotoxic, oxidative, and inflammatory responses relative to spherical particle controls in an epithelial inhalation model. By combining controlled fabrication with comparative biological evaluation, the results show that fiber-shaped MNPs can produce materially different cellular responses than spherical particles under the tested conditions. Although the present study was limited to two polymers, one epithelial cell model, and an acute exposure window, the findings support more explicit consideration of particle morphology in the evaluation of airborne MNP hazards.

Future studies should build on this morphology-controlled framework in several directions. First, more physiologically relevant lung models should be used to determine whether the cytotoxic, oxidative, and inflammatory responses observed here translate to impaired epithelial barrier function. A549 cells provide a useful alveolar epithelial model for evaluating cytotoxicity, oxidative stress, membrane damage, and inflammatory signaling, but they do not fully reproduce the tight barrier phenotype of differentiated primary alveolar epithelial cells or specialized airway epithelial barrier models. Therefore, while the observed LDH release, ROS production, and SEM evidence of membrane deformation suggest compromised epithelial integrity, they do not directly demonstrate tight junction disruption. Future work using differentiated epithelial barrier systems, air–liquid interface cultures, and cocultures with immune cells should include direct measurements of transepithelial electrical resistance, paracellular permeability, and junctional markers such as ZO-1, occludin, and claudins.

In addition, future studies should directly characterize fiber behavior in complete exposure medium using microscopy-based and time-resolved approaches to assess dispersion, aggregation, and sedimentation under exposure conditions. Broader material libraries are also needed to distinguish the relative contributions of polymer chemistry, fiber length, diameter, aspect ratio, rigidity, and environmental aging to biological response. These directions would extend, rather than replace, the present study by applying the controlled fiber-fabrication approach to more exposure-relevant and mechanistically resolved systems. More broadly, this work provides a practical experimental basis for future studies of fiber-relevant inhalation exposures and morphology-dependent microplastic toxicity.

## Supplementary Material



## Notes

Materials and methods for nanofiber fabrication, suspension preparation, cell exposure, toxicity assays, SEM analysis, and statistical analysis; photographs and SEM images of electrospun sheets, cryomilled fibers, and dispersed fiber suspensions; representative SEM images of fragmented PS and PAN fibers; length–diameter correlation analysis; FTIR spectra of DMF, pristine polymers, and isolated fiber MNPs; assay interference considerations (PDF)
