# Correction to “Engineered PS and PAN Microplastic Fibers Elicit Stronger A549 Epithelial Responses than Spherical Polystyrene Particles”

**DOI:** 10.1021/acs.chemrestox.6c00471

**Published:** 2026-09-22

**Authors:** Sewoon Kim, David M. Cwiertny, Dongkeun Lee, Jong Sung Kim, Jon A. Doering, Qinglin Wu

The funding acknowledgment was omitted from the originally published article. The correct Acknowledgment is as follows:

“This research was supported in part by the National Institute of Environmental Health Sciences (NIEHS) through the University of Iowa Environmental Health Sciences Research Center (EHSRC), NIEHS/NIH P30 ES005605.”

This correction does not affect the results or conclusions of the article.

