# Supplementary material for: Engineered PS and PAN Microplastic Fibers Elicit Stronger A549 Epithelial Responses than Spherical Polystyrene Particles
Source: Chem Res Toxicol. 2026 Aug 5;39(8):1609–16. doi: 10.1021/acs.chemrestox.6c00243 (PMC13488610; doi:10.1021/acs.chemrestox.6c00243)
Supplement: Supplementary file 1 [file tx6c00243_si_001.pdf]

Supporting Information for:

Engineered PS and PAN Microplastic Fibers Elicit Stronger A549 Epithelial Responses than Spherical Polystyrene Particles

Sewoon Kim[a*], David M. Cwiertny[b,c], Dongkeun Lee[d], Jong Sung Kim[d], Jon A. Doering[e], Qinglin Wu[f]

[a]*Department of Civil and Environmental Engineering, Louisiana State University, Baton Rouge, LA 70803, USA*

[b]*Department of Civil and Environmental Engineering, University of Iowa, 4105 Seamans Center, Iowa City, IA 52242, USA*

[c]*Department of Chemistry, University of Iowa, E331 Chemistry Building, Iowa City, IA 52242, USA*

[d]*Department of Occupational and Environmental Health, College of Public Health, University of Iowa, Iowa City, IA 52242, USA*

[e]*Department of Environmental Sciences, College of the Coast & Environment, Louisiana State University, Baton Rouge, LA 70803, USA*

[f]*School of Renewable Natural Resources, LSU AgCenter, Baton Rouge, LA 70803, USA*

**Corresponding author:*
E-mail: sewoon.kim@lsu.edu *(S. Kim)*

I. Materials and Methods

Chemicals. Polystyrene (PS, Mw = 192,000) and polyacrylonitrile (PAN, Mw = 150,000) were purchased from Sigma-Aldrich and used as the primary polymeric materials for fiber fabrication. N,N-Dimethylformamide (DMF, 99.8%) were obtained from Sigma-Aldrich as solvent for electrospinning. All chemicals were used without further purification. PS spheres with two different diameters (50 nm and 1 μm) were purchased from Polysciences, Inc. and used as spherical particle controls for comparison with polymeric fiber MNPs.

Nanofiber sheets preparation. Nanofiber sheets were synthesized using an electrospinning system under controlled environmental conditions. Polymer solutions were prepared by dissolving PS or PAN in DMF at a concentration of 20 % (wt.) for PAN and 10 % (wt.) for PS, followed by stirring at 60°C for 20 h. Electrospinning was conducted at an applied voltage of 15 kV, a flow rate of 0.5 mL/h, relative humidity of 20-25% with 20-25°C of chamber temperature, and a needle-to-collector distance of 10 cm. Electrospun nanofibers were collected on aluminum foil and dried under vacuum for overnight to evaporate remained solvent that might affect toxicity evaluation before further processing.

Preparation of Polymeric Fiber MNP Suspensions. Polymeric materials are generally difficult to disperse in aqueous media due to their hydrophobicity and low density. Therefore, polymeric fiber MNP suspensions were prepared using serum proteins as dispersants, following our previously reported method.[S1] Briefly, 1 mg of each polymeric fiber was dispersed in 1 mL of DI water containing 3% (v/v) fetal bovine serum (FBS) and sonicated for 10 min using a probe sonicator (QSonica, Newtown, CT, USA) to disrupt agglomerates. The resulting stock suspensions were diluted with Roswell Park Memorial Institute (RPMI) 1640 medium (Corning Inc., Corning,

NY, USA) supplemented with 10% FBS, 1% penicillin (100 units/mL), and 1% streptomycin (100 μg/mL). Working suspensions were sonicated for an additional 10 min immediately prior to cell exposure to ensure homogeneity. This FBS-assisted sonication protocol was used to minimize fiber aggregation before exposure. SEM analysis of the prepared fiber suspensions confirmed predominantly discrete, well-separated fibers after cryomilling, sonication, and filtration; however, time-resolved dispersion stability in complete exposure medium over the full 24 h exposure period was not directly quantified.

Cell Culture and Exposure Conditions. The human alveolar epithelial A549 cell line (ATCC, Manassas, VA, USA) was used as an in vitro pulmonary model. Cells were maintained in RPMI 1640 medium supplemented with 10% FBS, 1% penicillin, and 1% streptomycin at 37°C in a humidified incubator with 5% CO_2. Cells were seeded into 96-well culture plates at a density of 1×10^5 cells/mL and incubated for 24 h to allow cell attachment. Upon reaching approximately 85% confluency, cells were exposed to polymeric fiber MNPs or PS spheres (50 nm and 1 μm; Polysciences, Inc., Warrington, PA, USA) at final concentrations of 12.5, 25, and 50 μg/mL for 24 h. Mass concentration was used as the primary exposure metric because it enabled direct preparation and comparison of polymer suspensions at defined exposure levels. We recognize that this metric does not fully normalize differences in particle number or surface area between high-aspect-ratio fibers and spherical particles.

Cell Viability Assay. Cell viability was evaluated using the Alamar Blue assay (Sigma-Aldrich, St. Louis, MO, USA) following the manufacturer's instructions. After 24 h exposure, the culture medium was removed and cells were washed twice with pre-warmed Dulbecco's phosphate-buffered saline (DPBS). Fresh culture medium containing Alamar Blue reagent (final concentration: 50 μM) was added, and cells were incubated for 1 h at 37°C. The supernatant was

collected in amber tubes and centrifuged at 19,500 × g for 20 min at 4°C to remove suspended MNPs. Fluorescence was measured at excitation/emission wavelengths of 560/590 nm using a microplate reader (SpectraMax ID5, Molecular Devices, San Jose, CA, USA).

Intracellular Reactive Oxygen Species (ROS) Measurement. Intracellular ROS generation was measured using 5-(and-6)-carboxy-2′,7′-dichlorodihydrofluorescein diacetate (carboxy-H_2DCFDA; Invitrogen, Carlsbad, CA, USA). After exposure, cells were washed twice with pre-warmed DPBS and incubated with carboxy-H_2DCFDA (20 μM final concentration) in Hank's Balanced Salt Solution (HBSS) containing Ca^{2+} and Mg^{2+} for 45 min at 37°C in the dark. Cells were subsequently washed twice with DPBS, and 100 μL of HBSS was added to each well. Fluorescence intensity was measured at excitation/emission wavelengths of 485/535 nm using a microplate reader.

Lactate dehydrogenase (LDH) and IL-8 Measurements. Cell culture supernatants collected after exposure were first centrifuged at 700 × g to remove floating cells. The supernatants were then centrifuged at 19,500 × g for 20 min at 4°C to remove residual MNPs. Cytotoxicity was assessed by measuring LDH release using an LDH assay kit (Roche Diagnostics, Mannheim, Germany) according to the manufacturer's instructions, with absorbance measured at 490 nm. Pro-inflammatory responses were evaluated by quantifying interleukin-8 (IL-8) concentrations in the supernatants using a human IL-8 ELISA kit (Invitrogen, Carlsbad, CA, USA), with absorbance measured at 450 nm.

SEM Analysis of Cell–Fiber Interactions. Following 24 h exposure to MNPs, cell culture medium was removed and cells were washed twice with pre-warmed DPBS. Cells were fixed with 2% glutaraldehyde (Sigma-Aldrich, St. Louis, MO, USA) prepared in 0.1 M phosphate-buffered saline (PBS) for 1 h at room temperature. After fixation, cells were washed three times with 0.1 M

PBS for 10 min each and post-fixed with 1% osmium tetroxide for 5 min. Samples were washed again with PBS and dehydrated through a graded ethanol series (30%, 50%, 70%, 90%, and 100%). Dehydrated samples were dried by hexamethyldisilazane (HMDS; Sigma-Aldrich) evaporation, sputter-coated with a thin platinum layer, and imaged using scanning electron microscopy (SEM).

Statistical Analysis. All experiments were performed in biological triplicates ($n = 3$), and data are presented as mean ± standard deviation. Statistical analyses were conducted using SAS software (SAS Institute Inc., Cary, NC, USA). Differences between experimental groups were evaluated using one-way analysis of variance (ANOVA) followed by appropriate post hoc tests or Student's t-tests, as applicable. Statistical significance was defined as $p < 0.05$. Relationships between MNP physicochemical properties and toxicity endpoints were assessed using Pearson correlation coefficients and linear regression analysis.

II. Results and Discussion

Table S1. Evaluation of assay interference and IL-8 adsorption by polymeric particles and fibers.

Materials	Alamar blue interference (% of reagent-only control)	LDH interference (% of reagent-only control)	IL-8 recovery (% of particle-free IL-8 controls)[b]	IL-8 adsorption (%)
PS Spheres (50 nm)	100.8 ± 3.4[a]	95.8 ± 0.4	90.5 ± 1.6	9.5 ± 1.6
PS Spheres (1 μm)	99.8 ± 3.3	94.8 ± 0.6	91.3 ± 1.6	8.7 ± 1.6
PS Fiber	102.0 ± 3.2	96.5 ± 0.6	89.4 ± 1.0	10.6 ± 1.0
PAN Fiber	102.0 ± 3.3	96.8 ± 1.1	89.6 ± 1.4	10.4 ± 1.4

[a]Values are expressed as mean ± SD. Material-only controls for the Alamar Blue and LDH assays were performed at 50 μg/mL in the absence of cells and normalized to reagent-only controls.
[b]IL-8 recovery was determined by incubating recombinant IL-8 standard solution (100 pg/mL) with each material prior to ELISA analysis and normalizing to particle-free IL-8 controls.

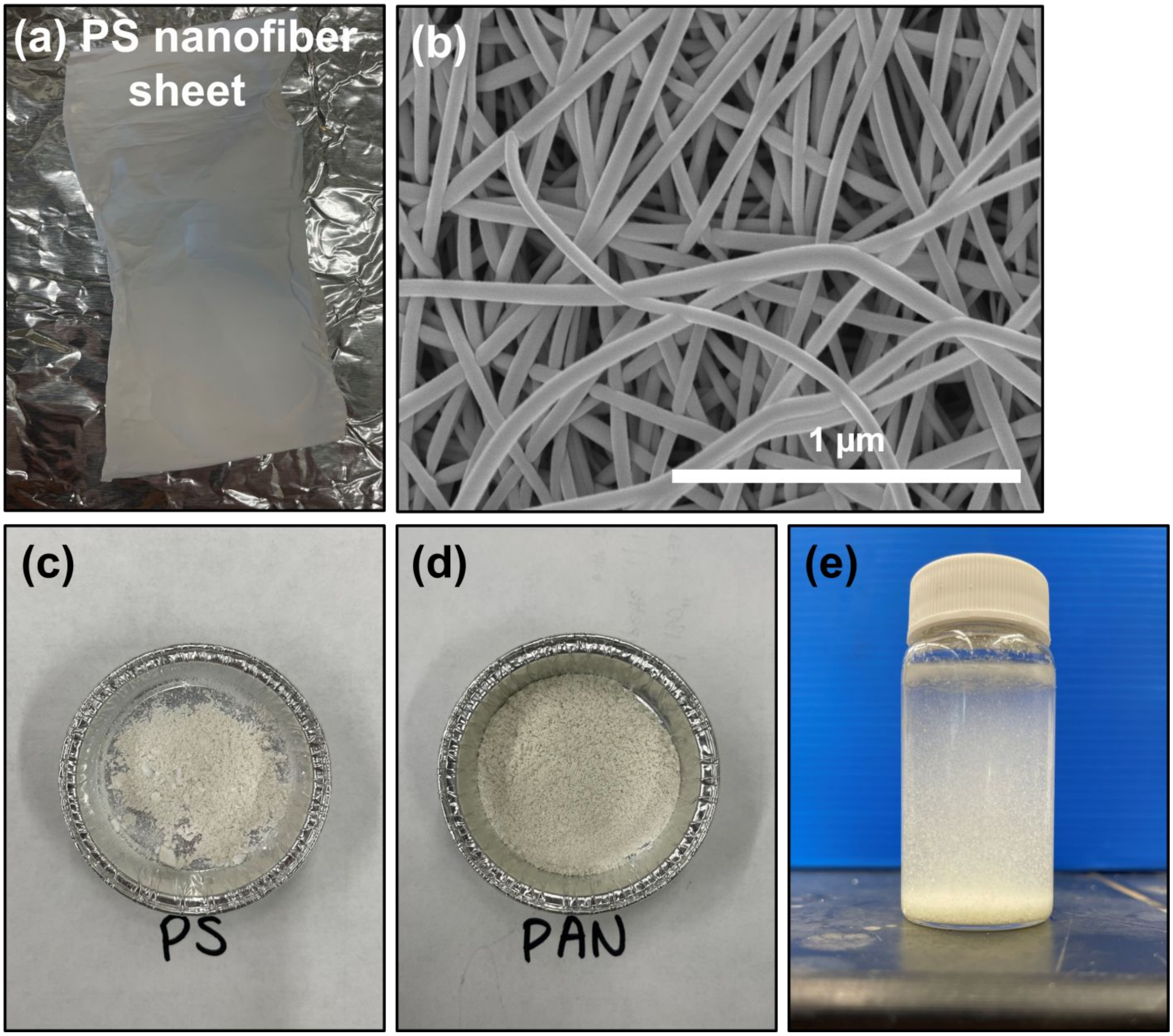

Figure S1. (a) Photograph of the electrospun PS nanofiber sheet and (b) corresponding SEM image showing a continuous, randomly entangled fibrous morphology. Electrospun PAN nanofiber sheets exhibited similar color and morphology and are therefore visually indistinguishable from PS sheets at the macroscopic scale. After cryomilling, both (c) PS and (d) PAN fibers appear as loose, powder-like aggregates. (e) photograph of a PS fiber suspension in DI water after probe sonication. Prior to use, the suspension was passed through a 30 μm woven nylon filter to remove aggregated or incompletely fragmented material and improve homogeneity for subsequent characterization and toxicity testing.

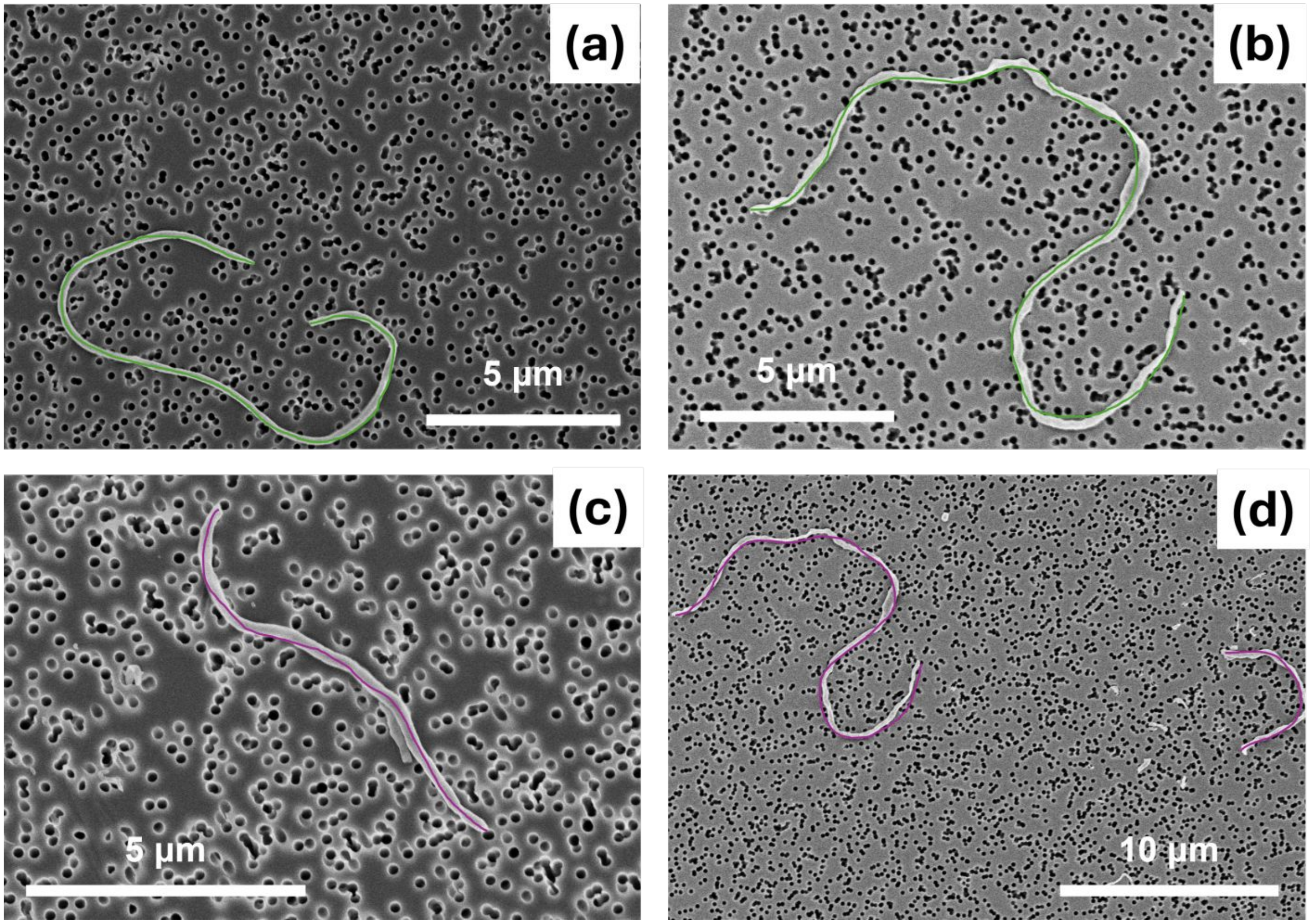

Figure S2. Representative SEM images of fragmented polymeric fibers collected on GTTP membrane filters. (a, b) PS fibers and (c, d) PAN fibers after cryomilling, probe sonication, and filtration are shown on the filter surface. The images reveal discrete, well-separated fibers with preserved cylindrical geometry and smooth surfaces, confirming efficient mechanical fragmentation without evidence of melting or deformation. Green (PS) and purple (PAN) traces indicate representative individual fibers selected for ImageJ-based dimensional analysis used to generate the statistics reported in Figure 1.

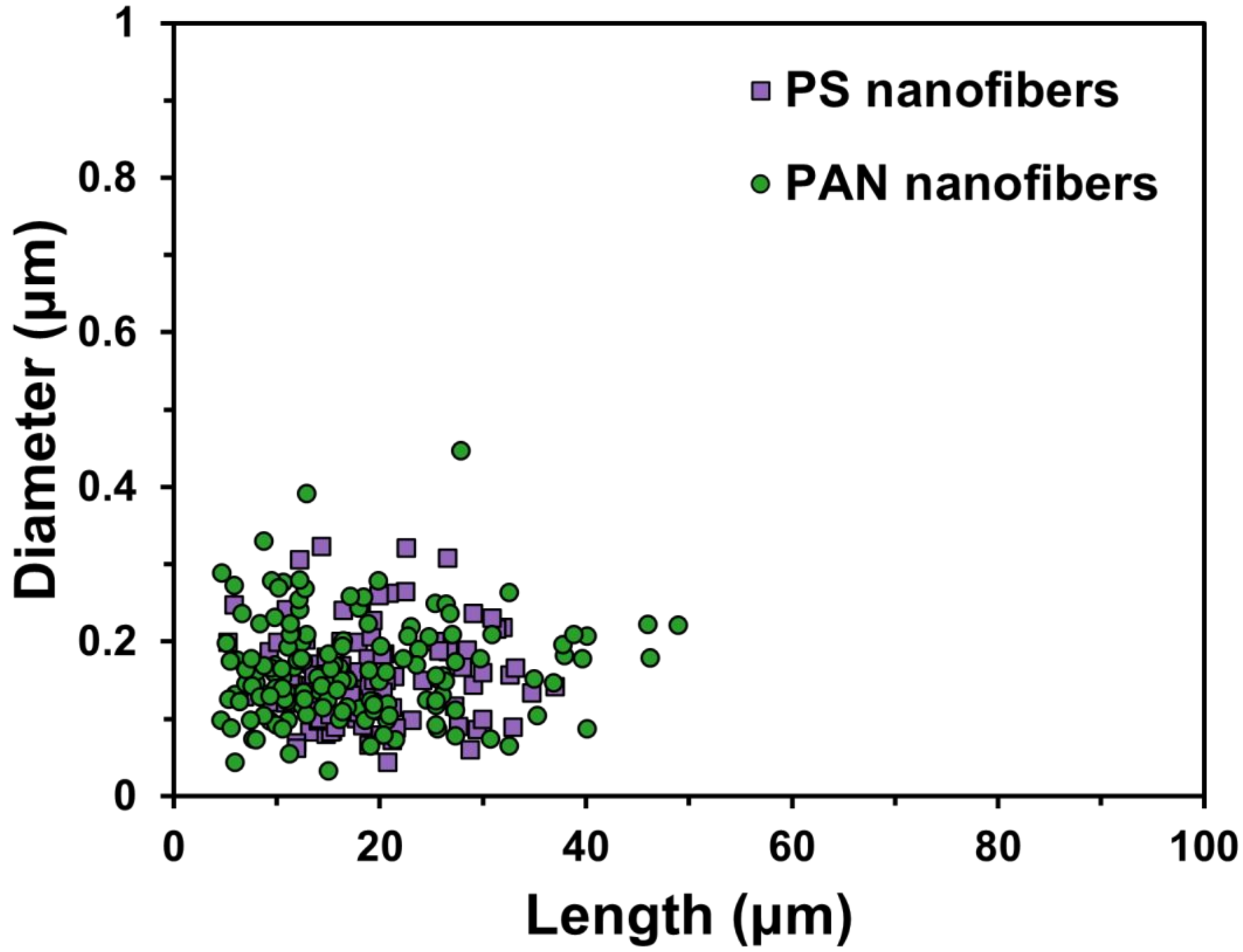

Figure S3. Length–diameter correlation of fragmented PS and PAN fibers. Each point represents an individual fiber measured from SEM images (n > 100 per polymer). PS (purple squares) and PAN (green circles) display overlapping distributions with no significant polymer-dependent differences in aspect ratio, indicating that the cryomilling, sonication, and filtration process yields reproducible, well-dispersed fibers. The absence of clustering suggests effective separation and minimal aggregation, supporting their suitability for controlled toxicological testing.

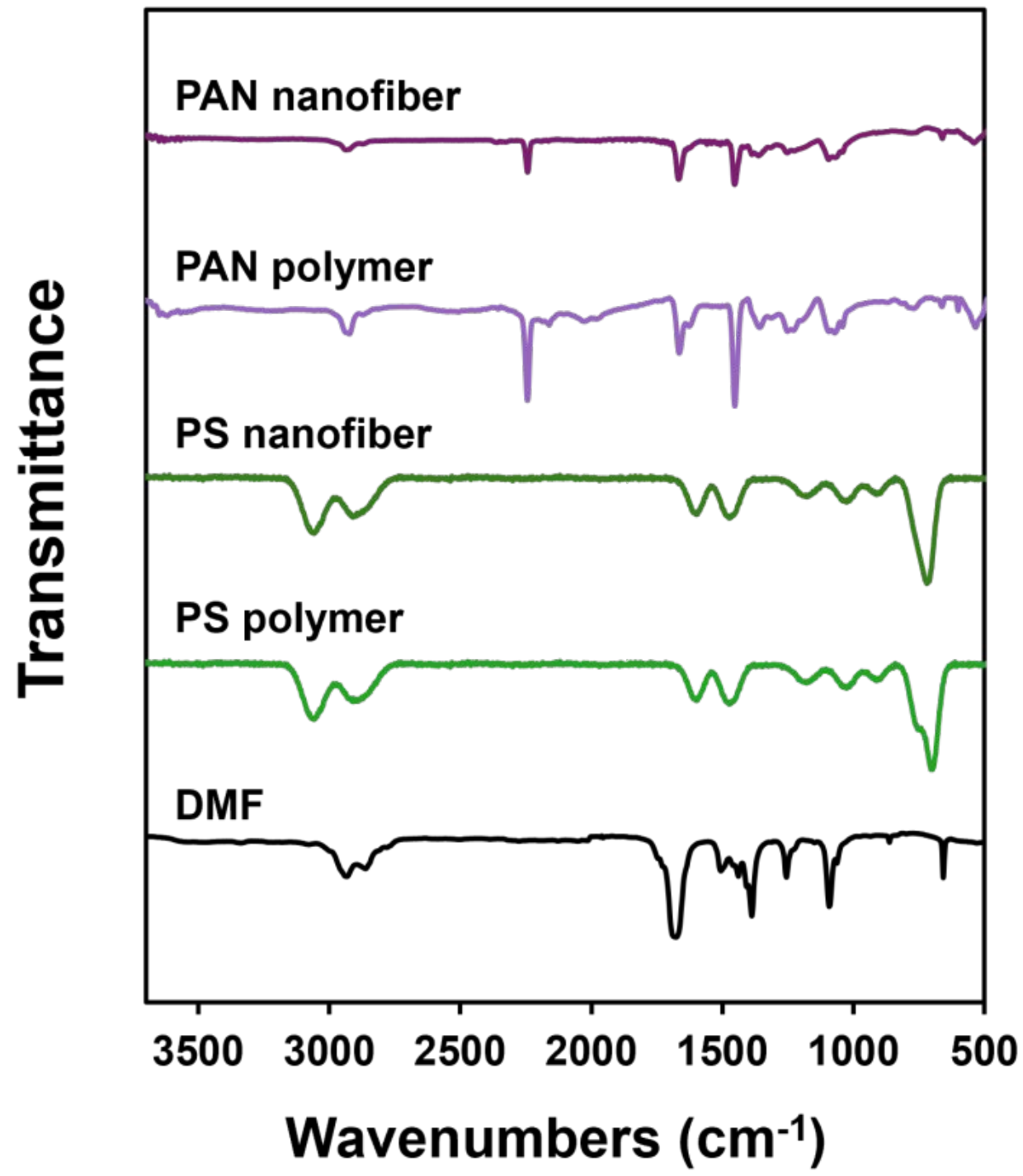

Figure S4. FTIR spectra of DMF, pristine PS and PAN polymers (vendor-supplied and no additional treatment), and isolated nanofibers obtained after frozen-cryomilling, sonication, and filtration of electrospun fiber mats. For both the solvents and polymeric nanofibers, all major spectral features match those previously reported in the literature for DMF,[S2,S3] PS,[S4,S5] and PAN.[S6-S8] This indicates that any residual DMF or unreacted polymer fragments remaining in the isolated fibers are below the detection limit of FTIR. For DMF, characteristic peaks include: ~2930 cm^{-1} and ~2860 cm^{-1} (aliphatic C–H stretching), ~1680 cm^{-1} (C=O stretching), ~1390 cm^{-1} (N–C–H bending), ~1255 cm^{-1} (C–N stretching), ~1090 cm^{-1} (C–N–C skeletal vibrations), and ~660 cm^{-1} (C–H out-of-plane). For PS, key peaks were observed at ~3060 cm^{-1} (aromatic C–H stretching), ~2900 cm^{-1} (aliphatic C–H), ~1600 cm^{-1} (aromatic C=C stretching), ~1500 cm^{-1} (aromatic ring vibration), and ~700 cm^{-1} (C–H out-of-plane bending of monosubstituted benzene). For PAN, characteristic peaks include ~2920 cm^{-1} (CH_2 stretching), ~2240 cm^{-1} (C≡N stretching), ~1730 cm^{-1} (C=O from oxidation or manufacturing), and ~1450 cm^{-1} (CH bending).

Reference

(S1) Lee, D. K.; Jeon, S.; Han, Y.; Kim, S. H.; Lee, S.; Yu, I. J.; Song, K. S.; Kang, A.; Yun, W. S.; Kang, S.-M.; Huh, Y. S.; Cho, W. S. Threshold rigidity values for the asbestos-like pathogenicity of high-aspect-ratio carbon nanotubes in a mouse pleural inflammation model. *ACS nano* **2018,** *12*(11), 10867-10879.

(S2) Malathi, M.; Sabesan, R.; Krishnan, S. IR carbonyl band intensity studies in N,N-dimethyl formamide and N,N-dimethyl acetamide on complex formation with phenols. *Current Science* **2004,** 86(6), 5

(S3) Ren, Y.-K.; Liu, S.-D.; Duan, B.; Xu, Y.-F.; Li, Z.-Q.; Huang, Y.; Hu, L.-H.; Zhu, J.; Dai, S.-Y. Controllable intermediates by molecular self-assembly for optimizing the fabrication of large-grain perovskite films via one-step spin-coating. *Journal of Alloys and Compounds* **2017**, 705, 205-210.

(S4) Campanale, C.; Savino, I.; Massarelli, C.; Uricchio, V. F. Fourier transform infrared spectroscopy to assess the degree of alteration of artificially aged and environmentally weathered microplastics. *Polymers* 2023, 15 (4), 911.

(S5) Betancourt-Galindo, R.; Cabrera Miranda, C.; Puente Urbina, B.; Castañeda-Facio, A.; Sánchez-Valdés, S.; Mata Padilla, J.; Garcia Cerda, L.; Perera, Y.; Rodríguez-Fernández, O. Encapsulation of silver nanoparticles in a polystyrene matrix by miniemulsion polymerization and its antimicrobial activity. *International Scholarly Research Notices* **2012**, 2012 (1), 186851.

(S6) Karbownik, I.; Fiedot, M.; Rac, O.; Suchorska-Woźniak, P.; Rybicki, T.; Teterycz, H. Effect of doping polyacrylonitrile fibers on their structural and mechanical properties. *Polymer* **2015**, 75, 97-108.

(S7) Jenab, A.; Roghanian, R.; Ghorbani, N.; Ghaedi, K.; Emtiazi, G. The efficacy of electrospun PAN/Kefiran nanofiber and kefir in mammalian cell culture: promotion of PC12 cell growth, anti-MCF7 breast cancer cells activities, and cytokine production of PBMC. *International journal of nanomedicine* **2020**, 717-728.

(S8) Tas, S.; Kaynan, O.; Ozden-Yenigun, E.; Nijmeijer, K. Polyacrylonitrile (PAN)/crown ether composite nanofibers for the selective adsorption of cations. *RSCAadvances* **2016**, 6 (5), 3608-3616.
